# A novel MoClo-mediated intron insertion system facilitates enhanced transgene expression in *Chlamydomonas reinhardtii*

**DOI:** 10.3389/fpls.2025.1544873

**Published:** 2025-03-07

**Authors:** Moritz Aschern, Jochem Braad, Alfonsina Milito, David Alzuria, Jae-Seong Yang

**Affiliations:** ^1^ Centre for Research in Agricultural Genomics (CRAG), CSIC-IRTA-UAB-UB, Barcelona, Spain; ^2^ Doctoral Program of Biotechnology, Faculty of Pharmacy and Food Sciences, Universitat de Barcelona, Barcelona, Spain

**Keywords:** synthetic biology, *Chlamydomonas reinhardtii*, transgene expression, intron-mediated enhancement (IME), Modular Cloning (MoClo), microalgal biotechnology

## Abstract

The *Chlamydomonas* Modular Cloning (MoClo) toolkit allows for straightforward and flexible construction of genetic modules for gene expression in the microalgal model species, fostering developments in algal biotechnology. Efficiently expressing transgenes from the nuclear genome of *C. reinhardtii* requires the proper insertion of introns throughout the respective gene, as it can substantially enhance the gene expression. To facilitate synthetic biology approaches in this microalga, we developed a novel strategy for intron insertion into synthetic DNA fragments. Our method aligns with current MoClo standards, and its feasibility is demonstrated by assembling genes of various lengths and successfully expressing them in *C. reinhardtii*. Examples include enhanced *NanoLuc* expression with increased intron numbers, a fungal luciferase enabling bioluminescence in *C. reinhardtii*, and a fungal tryptophan decarboxylase.

## 1 Introduction

Shifting from a petroleum-based economy to a circular bioeconomy is essential for tackling global environmental challenges. Bioeconomy relies on renewable biological resources to produce food, materials, and energy while vastly reducing carbon emissions. This transition requires innovative biotechnological tools to make biological systems more efficient and suitable for industrial use (Shirvani, 2024). Microalgae are promising organisms to be used for sustainable biotechnology due to their ability to produce biomass and carbon-based compounds from CO_2_ whilst generating energy required for the underlying cellular processes via photosynthesis. The green alga *Chlamydomonas reinhardtii* has particularly good prospects due to its ease of culture and scalability thereof, availability of advanced genetic engineering tools and the abundance of genomic and metabolic knowledge (Findinier and Grossman, 2023; Milito et al., 2023).

However, to effectively use *C. reinhardtii* in biotechnology, it is crucial to achieve high levels of heterologous gene expression. One important factor that can greatly influence gene expression in this microalga is the systematic incorporation of endogenous introns into transgenes (Lumbreras et al., 1998; Eichler-Stahlberg et al., 2009). This approach leverages a phenomenon termed intron-mediated enhancement (IME), where the presence of introns within a gene significantly boosts its expression levels. Intron addition mimics the natural structure of endogenous genes of C. *reinhardtii*, that has a particularly high density of introns with on average 7.3 introns per gene with an average exon length of just 240 nucleotides (Merchant et al., 2007). Systematic analyses of IME have identified optimal insertion sites within genes and recommended maintaining exon lengths below 500 bp to achieve optimal expression levels in this organism (Baier et al., 2018). Further study revealed that native introns vary in IME effectiveness based on their sequences, with the top five highly effective IME-causing introns ranging from 140 to 250 nucleotides in length (Baier et al., 2020). Considering these facts, repetitive introns account for approximately 20% to 30% of the heterologous genes required for microalgal expression.

Efforts to modularize repetitive genetic elements are advancing synthetic biology by enabling the standardized sharing of components and the rapid, reproducible construction of genetic circuits (Bird et al., 2022). One notable approach is the MoClo system, a standardized DNA assembly method which employs type IIS restriction enzymes and a DNA ligase to precisely join DNA fragments in a single reaction (Weber et al., 2011). A dedicated toolkit for *C. reinhardtii* offers a powerful solution for assembling complex genetic constructs from standardized parts in a flexible way and facilitate systematically testing a multitude of different gene expression elements (Crozet et al., 2018). The toolkit supplies standard parts (e.g. promoters, coding sequences, terminators, etc.) referred to as level 0 parts, which can be used in assemblies to form transcriptional units referred to as level 1 constructs which again can be assembled into multigenic constructs termed level 2 constructs. Despite its advantages, this MoClo toolkit does not include streamlined methods for flexibly adding introns to genes prior to the assembly of expression constructs. To fully exploit IME for improving gene expression, advanced MoClo-compatible tools specifically designed to facilitate intron integration are needed.

In this study, we developed a new method for flexibly incorporating introns into synthetic genes using the MoClo syntax. As transgenes need to resemble the *C. reinhardtii* codon usage to be expressed to desirable levels, the gene of interest is usually codon optimized and introns are spread *in silico* throughout the coding sequence before the DNA is synthesized. Synthesis of large genes is generally complicated by their high GC content and repetitive sequences, requiring the gene to be split up into different parts to be assembled into a level 0 backbone of the MoClo system (Schroda, 2019). Our methodology leverages this gene design requirement to achieve greater flexibility in level 0 coding sequence assemblies. The previously codon optimized gene is split into exons which are then assembled with introns, resulting in functional genes. The approach is fully compatible with existing MoClo standards and allows different introns to be flexibly added into the same gene sequences during the assembly process of level 0 modules. We demonstrate the effectiveness of this method by assembling and expressing several genes in *C. reinhardtii*, including two bioluminescent reporter genes and a tryptophan decarboxylase that directs higher amounts of tryptophan towards auxin biosynthesis via generation of tryptamine. Our concept simplifies intron integration into transgenes, providing a valuable tool for advancing algal biotechnology and synthetic biology.

## 2 Materials and methods

### 2.1 *Chlamydomonas reinhardtii* cultivation and transformation

*C. reinhardtii* LMJ.RY0402.148523 (hereafter termed LM8523 for conciseness), a descendant of strain CC-4533 (cw15, mt-) harboring an insertional disruption of the *SRTA* histone deacetylase gene (Neupert et al., 2020), was obtained from the Chlamydomonas Library Project (Li et al., 2019). Cells were routinely maintained on Tris-Acetate-Phosphate (TAP) agar (Gorman and Levine, 1965) under mixotrophic conditions at a constant light intensity of 10 µmol photons m^-2^ s^-1^. Liquid cultivations were performed in shake flasks if not specified otherwise, using TAP medium at a constant light intensity of approx. 60 µmol photons m^-2^ s^-1^, 24°C and 100 rpm.

Cells were transformed through electroporation (Milito et al., 2024) using 8x10^6^ cells, 500 ng of linearized DNA and 20 µg salmon sperm DNA (Fisher Scientific). Cells were regenerated overnight in TAP medium and plated on TAP agar containing 20 µg/mL hygromycin B (Gibco). Plates were incubated under low light at room temperature until colonies appeared.

Single transformants were cultivated for reporter activity screening by inoculating single colonies into 200 µL TAP medium in a 96-well microtiter plate, followed by an incubation for 3 days. Selected single transformants were afterwards cultured in 6- or 12-well plates for further mutant characterization. Pools of transformants were cultivated for analysis by scraping off colonies from agar plates using TAP medium and a Drigalski spatula, pooling more than 1000 colonies per construct and experiment. The pooled cells were incubated overnight in 25 mL TAP medium.

### 2.2 Transgene design and cloning

PCR reactions were performed using NZYProof DNA polymerase (NZYtech). Plasmid extractions were performed with the NucleoSpin Plasmid EasyPure kit (Macherey-Nagel), DNA was extracted from agarose gels with the NucleoSpin Gel- and PCR Clean-up kit (Macherey-Nagel). Gibson Assembly master mix was acquired from the Protein Technologies Unit of the Centre for Genomic Regulation. Restriction enzymes and other enzymes for molecular biology applications used were obtained from New England Biolabs. Primers were ordered from Integrated DNA Technologies and those used for cloning are listed in **Supplementary Table 1**, synthesized DNA fragments were ordered from Twist Bioscience and are listed in **Supplementary Table 2**. MoClo backbone plasmids from the pICH and pAGM series (Weber et al., 2011) were acquired via Addgene. Level 0 parts of the Chlamydomonas MoClo toolkit (Crozet et al., 2018) were acquired from the Chlamydomonas Resource Center. If not described otherwise, all previously mentioned reagents and kits were used according to the manufacturer’s instructions. MoClo assemblies were carried out as described by Crozet et al. (2018), with a modified temperature program (50 cycles of digestion at 37°C for 5 min and ligation at 16°C for 5 min, followed by one final digestion step at 37°C for 60 min and one enzyme inactivation step at 80°C for 20 min).

The level -1 backbone vector pCM-1 was made via Gibson assembly from pICH47732. The vector was cut with DraIII and the large backbone fragment was extracted from an agarose gel. The *lacZ* cassette was amplified without BbsI recognition sites, adding overhang sites complementary to the backbone before being inserted therein.

The level 0 backbone vectors pICH41258_B3A and pICH41258_B3B were made via Gibson assembly from pICH41258 (Weber et al., 2011). Each plasmid was amplified in two fragments, changing the downstream BbsI overhang sequence in pICH41258_3A and the upstream BbsI overhang sequence in pICH42258_3B to ACGA. The two fragments each were then joined to yield the final plasmids.

DNA fragments encoding either the first or the second intron of the ribulose-1,5-bisphosphate carboxylase/oxygenase (RuBisCO) small subunit 2 for level -1 parts were designed by adding the appropriate 4 bp overhang up- and downstream of the intron sequence that matches their insertion site in the gene, followed by a BbsI recognition site so that upon digestion, the previously mentioned overhangs were generated. Additionally, up- and downstream thereof, overhang sequences and a BsaI recognition site were added, revealing GGAG and CGCT overhangs upon digestion up- and downstream, respectively, that allow for insertion into pCM-1. Level -1 parts coding for introns were assembled from 200 fmol DNA fragments, coding for the intron and 100 fmol backbone pCM-1 in a MoClo reaction.

DNA fragments encoding the *Neonothopanus nambi* luciferase Luz_v4 (Shakhova et al., 2024) and the *Psilocybe cubensis* tryptophan decarboxylase PsiD (UniProt: P0DPA6; Fricke et al., 2017) were designed by reverse translating their amino acid sequence using the optimal codon usage of *C. reinhardtii*. For NanoLuciferase (NanoLuc), a previously codon-optimized sequence was used (Crozet et al., 2018). The sequences were split into 2 (NanoLuc and *nn*Luz_v4) and 4 (*Pc*PsiD) parts, flanked by BbsI recognition sites, so that upon digestion, AATG and AGGT overhangs were generated at the upstream part of the first fragments and the downstream part of the last fragments, respectively. This allowed cloning the level 0 parts as B3 modules of the MoClo syntax. All other ends of the fragments, located at internal sections of the genes, were flanked by BbsI recognition sites, so that overhangs were generated upon digestion allowing seamless insertion of level -1 intron parts. Only optimal insertion sites (Baier et al., 2018) were chosen for intron insertion and exon length was between 234 bp and 432 bp. Finally, DNA fragments were synthesized.

Level 0 parts for the previously mentioned genes were assembled from 50 fmol of each gene fragment, coding for exons, 50 fmol of each level -1 part, coding for introns and 50 fmol of the backbone in a MoClo assembly, as described in **Table 1**. Level 0 parts encoding a flexible GGSGGR linker (pCM0-Link) and an alternative flexible GGSGGR linker (pCM0-Link_alt) that corrects the frameshift in wrongly designed B3 parts, such as pCM0-061-CrNanoLuc, were assembled via annealing of oligonucleotides. The annealed DNA was cloned into pAGM1299 for B4 parts (Weber et al., 2011) in a MoClo assembly.

**Table 1:** Assembly of level 0 parts from DNA fragments, level -1 parts, and a backbone.

Name	DNA fragments used	Level -1 part(s) used	Backbone used
pCM0-NanoLuc_i1	Fragment_NanoLuc_1 (B3), Fragment_NanoLuc_2 (B3)	pCM-1_i1_001	pICH41258
pCM0-*nn*Luz_v4_i1	Fragment_Luz_1 (B3), Fragment_Luz_2 (B3)	pCM-1_i1_001	pICH41258
pCM0-*Pc*PsiD_i1	Fragment_PsiD_1 (B3), Fragment_PsiD_2 (B3), Fragment_PsiD_3 (B3), Fragment_PsiD_4 (B3)	pCM-1_i1_002, pCM-1_i1_003, pCM-1_i1_004	pICH41258
pCM0-NanoLuc_i2	Fragment_NanoLuc_1 (B5), Fragment_NanoLuc_2 (B5)	pCM-1_i2_001	pAGM1301

Level 2 expression vectors were cloned using pMBS810 as a backbone, allowing for a rapid one-step assembly directly from level 0 parts (Niemeyer and Schroda, 2022) in a MoClo assembly. The level 0 parts used in this study are described in **Supplementary Table 3**.

During cloning, all plasmids were transformed into either chemically competent *Escherichia coli* TOP10 via heat-shock or into electrocompetent *E. coli* DH10B via electroporation using standard conditions. Competent cells were prepared in-house. All plasmids produced were quality controlled by restriction digest and confirmed by Sanger sequencing (Capillary Sequencing Core Facility, Centre for Research in Agricultural Genomics).

### 2.3 RNA extraction and analysis by real-time qPCR

RNA was extracted using the Maxwell^®^ RSC Plant DNA Kit (Promega). Approximately 3x10^7^ cells were harvested, pellets were resuspended in 400 µL 1-thioglycerol/homogenization solution and mixed with 200 µL lysis solution, before processing them according to the manufacturer’s instructions.

1000 ng RNA were used for cDNA synthesis with the Maxima H Minus First-Strand cDNA Synthesis Kit, with prior dsDNase treatment (Thermo Fisher Scientific), according to the manufacturer’s instructions. For quantification, 20 µL reactions were prepared with LightCycler 480 SYBR Green I Master Mix (Roche), amplifying 10 ng cDNA per reaction in a QuantStudio 6 Pro thermocycler (Thermo Fisher Scientific). Expression of the transformed constructs was measured using primers binding in the exon regions of *mVenus*, flanking the intron (qPCR_mVenus_F: 5’-CACCATCTTCTTCAAGGACGA-3’, qPCR_mVenus_R: 5’-TGTAGTTGTACTCCAGCTTGT-3’; successfully tested for amplification efficiency as demonstrated in **Supplementary Figure 2B**) and normalized to the expression of *GBLP* as a housekeeping gene (F: 5’- CAAGTACACCATTGGCGAGC-3’, R: 5’- CTTGCAGTTGGTCAGGTTCC-3’; Petroutsos et al., 2016). Differences in gene expression were calculated via the 2^-ΔΔCt^ method (Livak and Schmittgen, 2001), comparing expression of the intronized constructs to their non-intronized counterparts.

### 2.4 Protein extraction, SDS-PAGE, and In-Gel detection of NanoLuc

Approximately 5x10^7^ cells were harvested and pellets were resuspended in 500 µL modified RIPA buffer (50 mM Tris-HCl (pH 7.5), 150 mM NaCl, 1% v/v Triton X-100, 0.1% w/v SDS, 10 mM NaF, 1 mM EDTA), supplemented with 2% protease inhibitor cocktail for plant and cell tissue extracts (Sigma-Aldrich) and PMSF (Thermo Fisher Scientific) at 1 mM final concentration. Cells were then lysed on ice in an SFX 550 sonicator (Branson) at 20% duty cycle using a 3 mm tapered microtip, 3 times for 30 s each, with 1 min breaks in between each step. Cell debris was spun down at 16000 g for 10 mins at 4°C and protein concentration of the supernatant was determined using the Bradford Protein Assay (Bio-Rad) with 0.1 - 1.5 µg µL^-1^ BSA as a standard.

Subsequently, 15 µg protein of each sample were mixed with loading buffer (62.5 mM Tris-HCl (pH 6.8) 10% v/v glycerol, 2% w/v SDS, 0.02% w/v bromophenol blue), supplemented with 2-mercaptoethanol to a final concentration of 4% v/v and heated for 5 min at 95°C. Proteins were separated by SDS-PAGE (12% acrylamide) and NanoLuc was detected using the Nano-Glo^®^ In-Gel Detection System (Promega), according to the manufacturer’s instructions. After successful imaging, the detection reagent was washed off the gel with deionized water and protein fixation was achieved in the gel by incubation for 15 min with fixation solution (40% v/v ethanol, 10% v/v acetic acid). Proteins were stained using the QC Colloidal Coomassie Stain (Bio-Rad) overnight, de-stained 3 times for 1 h each with deionized water and imaged. All images were taken using Amersham ImageQuant 800 (GE Healthcare).

### 2.5 Determination of NanoLuc activity

NanoLuc activity was measured using the Nano-Glo^®^ Luciferase Assay System (Promega), adapting the manufacturer’s protocol to *C. reinhardtii* cell suspensions (Niemeyer et al., 2023). For measuring single transformant cultures, 50 µL culture was used directly in the experiment. For pooled cultures, 1.5x10^7^ cells were harvested and resuspended in 3 mL TAP medium, 50 µL of this cell suspension was then used for the experiment. NanoLuc activity was measured by mixing 50 µL culture with 50 µL assay reagent in a white 96-well plate, subsequently the measurement was performed at 460 nm with a SpectraMax M2 microplate reader (Molecular Devices) using an integration time of 500 ms. The signal of wild-type LM8523 cells was subtracted from the readings to account for the background signal. Chlorophyll fluorescence was measured using cultures of 100 µL per well of a black clear-bottom 96-well plate in a SpectraMax M2 microplate reader (excitation: 440 nm, emission: 680 nm). NanoLuc readings were normalized to chlorophyll fluorescence measurements to account for differences in cell density.

### 2.6 Determination of Luz activity

Approximately 1.5x10^7^ cells were harvested and washed with 300 µL PBS, before being resuspended in 300 µL *Renilla* Luciferase Assay lysis buffer (Promega). Proteins were extracted by vigorous mixing for 30 s and the mixture was centrifuged at 10000 g for 10 min at 4°C. The protein concentration in the supernatant was determined as previously described. For measurement of Luz activity, 40 µL of the supernatant were incubated in triplicate with either 10 µL DMSO or 10 µL 3-hydroxyhispidin (20 mM in DMSO; kindly supplied by Karen Sarkisyan). The measurements were performed at 530 nm in a Victor Nivo microplate reader (PerkinElmer) using an integration time of 500 ms. The signal of wild-type LM8523 cells was subtracted from the readings to account for the background signal, and readings were normalized to the protein concentration of the extracts.

### 2.7 Tryptamine extraction and quantification

Tryptamine was extracted from cells and quantified via HPLC as described by Ly et al. (2008), with several modifications. Briefly, 5x10^7^ cells were harvested, and the pellet was extracted with 750 µL of methanol by vigorous mixing for 3 min. After centrifugation at 16000 g for 10 mins, the supernatant was collected. The pellet was subjected to another round of extraction with 750 µL 50% (v/v) methanol, joining the collected supernatants. The mixture was evaporated to dryness and dissolved in 200 µL 50% (v/v) methanol. The culture supernatant of 5x10^7^ cells was firstly evaporated to dryness and then extracted via the same method. For quantification, the samples were separated by reversed-phase HPLC on an Atlantis dC18 column (3 µm, 3.9 x 150 mm; Waters), using an isocratic elution of 35% (v/v) solvent A (methanol) and 65% (v/v) solvent B (water with 0.3% (v/v) trifluoroacetic acid). The column was temperature-controlled at 20°C and operated at a flux of 0.5 mL/min, resulting in a pressure of approximately 190 bar. Detection occurred at 280 nm. Tryptamine (Sigma-Aldrich) and indole-3-acetic acid (IAA; Sigma-Aldrich) were used as analytical standards.

## 3 Results

### 3.1 An intron insertion approach that adds flexibility to transgene assemblies

**Figure 1:**
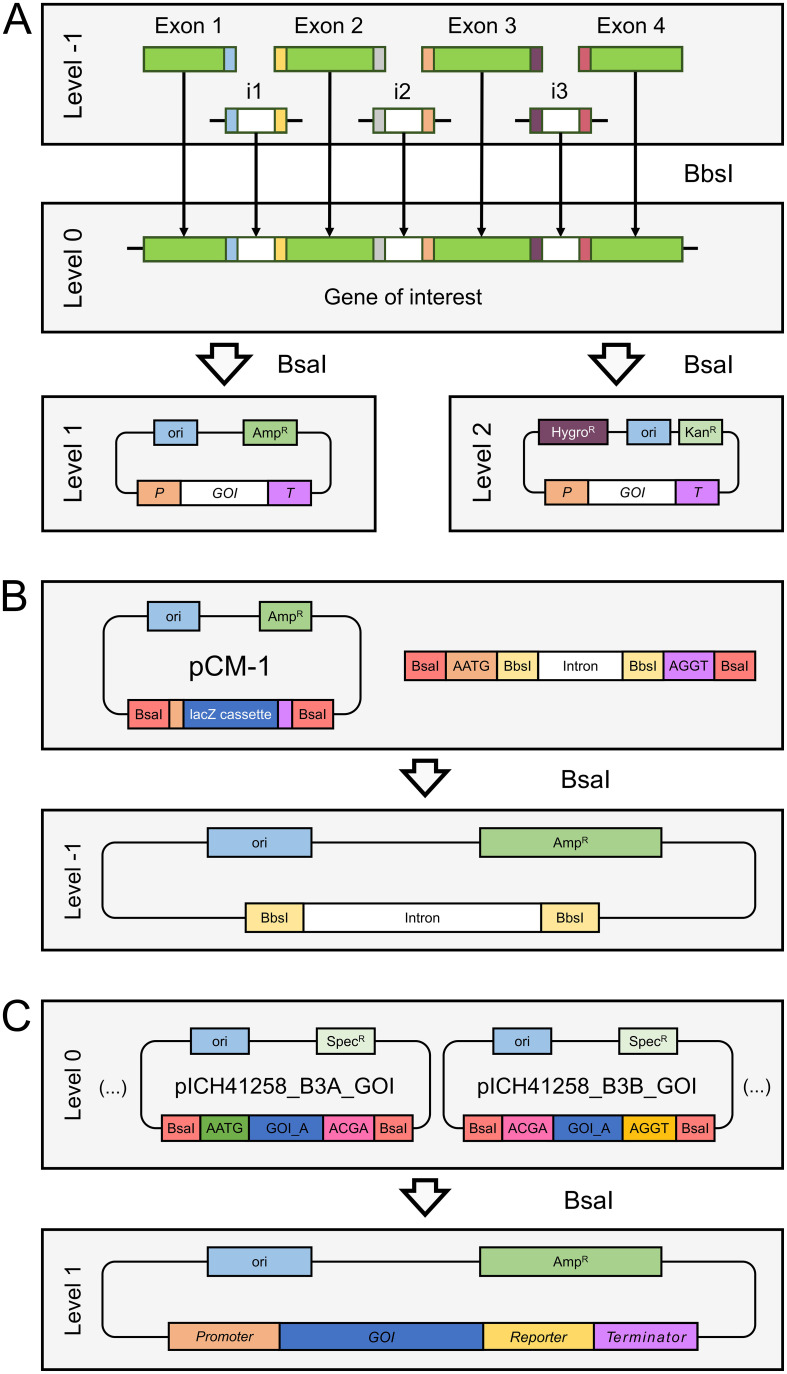
Schematic overview over the functioning of the MoInClo system. **(A)** Illustrative, simplified assembly of a gene of interest as a level 0 part from four exon gene fragments and three level -1 intron parts, resulting in the full-length gene on a plasmid backbone. This gene can subsequently be cloned into level 1 modules using the standard MoClo backbone plasmids or directly into level 2 devices, using the backbone plasmids of Niemeyer and Schroda (2022). Colored boxes represent overlapping sites that are exposed after restriction digest and allow for seamless cloning. P, Promoter; GOI, Gene of interest; T, Terminator; Ori, Origin of replication; Kan^R^, Bacterial kanamycin resistance cassette. **(B)** Cloning of a synthesized intron sequence into the backbone vector pCM-1 via its BsaI recognition site. Higher level clonings are enabled by the BbsI recognition site. Amp^R^: Bacterial ampicillin resistance cassette. **(C)** Exemplary application of the B3A and B3B plasmids. After successful cloning of a split GOI into these backbones, they allow for seamless assembly of the full-length sequence into a level 1 module. Spec^R^: Bacterial spectinomycin resistance cassette. The restriction enzyme needed for each step is indicated between the boxes.

In this work, we aim to add flexibility and ease to level 0 gene assemblies of the MoClo system by introducing level -1 vectors containing introns as an additional level that aligns with the currently existing syntax. According to our approach, termed MoInClo (Modular Intron Cloning), a transgene is split up into introns and exons equipped with BbsI cut sites that, upon digestion, reveal overhangs allowing for the seamless assembly of a fully functional CDS (**Figure 1A**). This allows for flexible insertion of different introns into the same gene without the need to use complicated cloning techniques to modify existing level 0 parts or re-purchase the synthetic gene. The resulting part is compatible with the MoClo framework and can then be used to assemble higher-level expression plasmids. We use the intron insertion sites recommended by Baier et al. (2018). Their work showed that introns flanked by guanine on both sides result in maximum splicing efficiencies (**Supplementary Figure 1A**) and furthermore revealed several combinations of nucleotides at the second position that result in strong IME (**Supplementary Figure 1B**). The exons used for the cloning of level 0 modules can be used directly as synthetic gene fragments, while the synthetic intron DNA can either be used directly or cloned into a level -1 backbone vector (**Figure 1B**). The latter, while being more labor-intensive, makes it possible to amplify the plasmid and reuse the same intron for future assemblies of other genes.

As assemblies of long genes with more than 3 or 4 introns are complicated due to the high number of fragments needed for their assembly, we supply two novel backbone vectors in B3 position of the MoClo syntax. The B3A and B3B plasmids are based on the B3 backbone vector pICH41258. They were modified to contain a site that, after successful assembly of the level 0 part, enables fusing two level 0 gene sequences into one functional CDS when assembled into an expression module (**Figure 1C**). The two parts are joined at the ACGA overlap, a common sequence found within codon-optimized transgenes that does not interfere with any other overhang generated during level 1 assembly.

### 3.2 Level 0 parts are easily assembled using MoInClo

**Figure 2:**
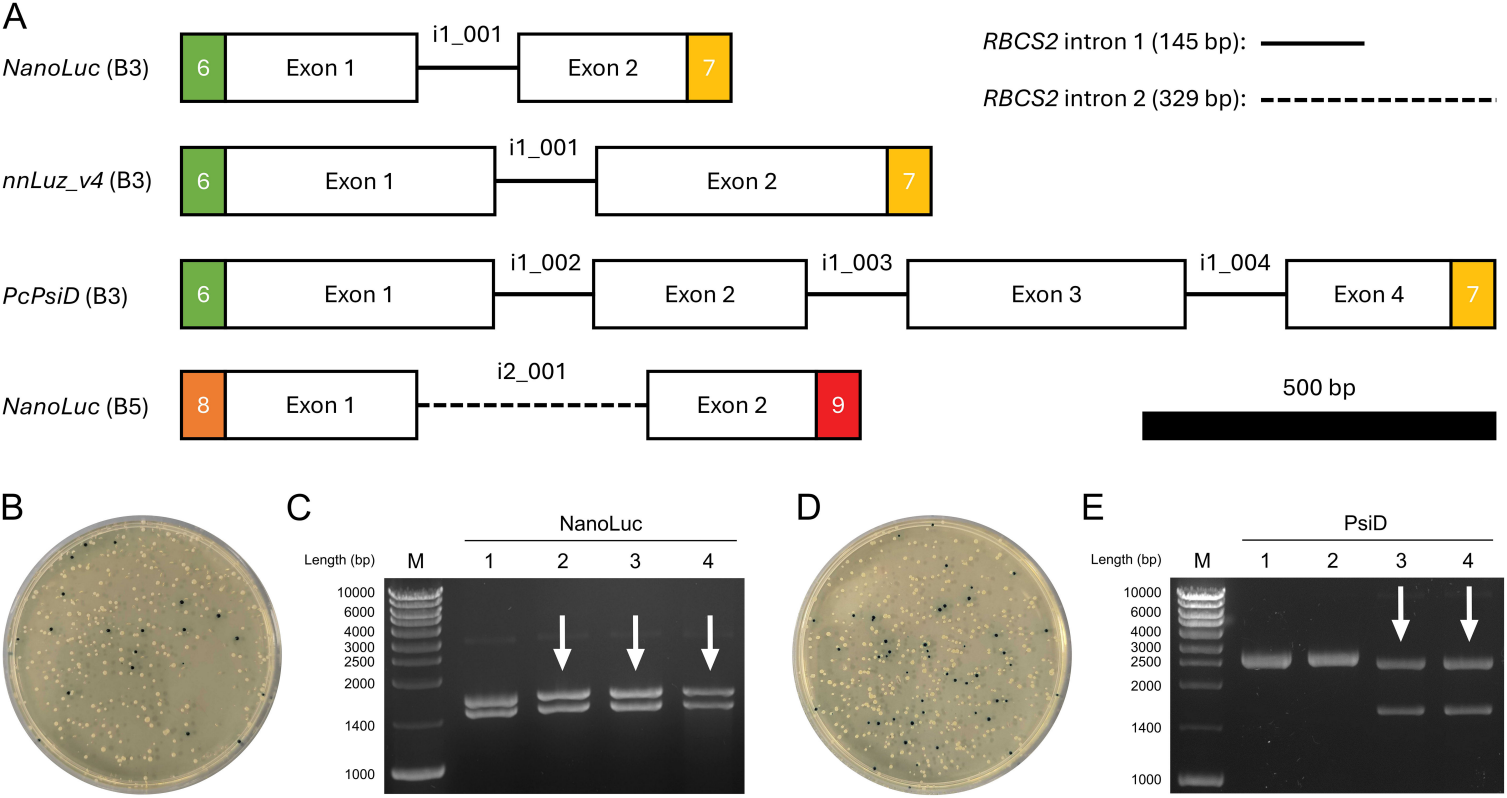
Cloning process of selected level 0 parts using MoInClo. **(A)** Schematic representation of the cloned level 0 parts used in this study. The color code for the fusion sites follows Crozet et al. (2018). Level -1 introns used for assembly are described for each part. *RBCS2* intron 1 and intron 2 are shown as lines and dashed lines, respectively. The 500 bp scale bar allows size comparison of the different parts. **(B)** Transformation of *E. coli* with the MoInClo assembly reaction of *NanoLuc* (B5) and blue-white screening after selection on spectinomycin. **(C)** Restriction digest of plasmids purified from 4 white *E. coli* transformants for *NanoLuc* (B5) using BlpI. The correctly assembled plasmid yields two fragments at 1460 bp and at 1633 bp upon digestion. **(D)** Transformation of *E. coli* with the MoInClo assembly reaction of *PcPsiD* (B3) and blue-white screening after selection on spectinomycin. **(E)** Restriction digest of plasmids purified from 4 white *E. coli* transformants for *PcPsiD* (B3) using BlpI. The correctly assembled plasmid yields two fragments at 1573 bp and at 2429 bp upon digestion. Correctly digested plasmids are marked by white arrows.

We used MoInClo to assemble level 0 parts of CDS from level -1 introns and exons that were supplied as synthetic gene fragments after codon-optimization. The method was validated by assembling four distinct parts, varying in length, position, and intron type (**Figure 2A**). *NanoLuc, Luz* and *PsiD* were intronized with RBCS2i1 in B3 position of the *Chlamydomonas* MoClo system. Additionally, *NanoLuc* was also intronized with RBCS2i2 and cloned in B5 position to serve as an intronized reporter gene for C-terminal gene fusions. Cloning generally had a relatively high success rate for the stated parts when assessed via blue-white screening. Two representative cloning plates are shown to outline cloning efficiency, one depicting the results of the three-fragment assembly of *NanoLuc* (**Figure 2B**) and the other displaying the results of the seven-fragment assembly of *PsiD* (**Figure 2D**). For both clonings, vast proportions of the colonies appeared white, hinting at correct insertion of the desired insert into the backbone. Due to the common occurrence of false positives in blue-white screenings, we conducted further analysis on four white colonies per construct using restriction digestion. The three-fragment assembly achieved a success rate of 75% correct clones (**Figure 2C**), while the seven-fragment assembly resulted in 50% correct clones (**Figure 2E**). These results indicate that the assembly process works with sufficiently high efficiency to reliably generate the desired parts, even with increasing assembly complexity. Finally, the full length of the inserts was successfully verified by Sanger sequencing for each part before being further assembled into expression constructs.

### 3.3 Assembly into functional transcriptional units driving NanoLuc expression

**Figure 3:**
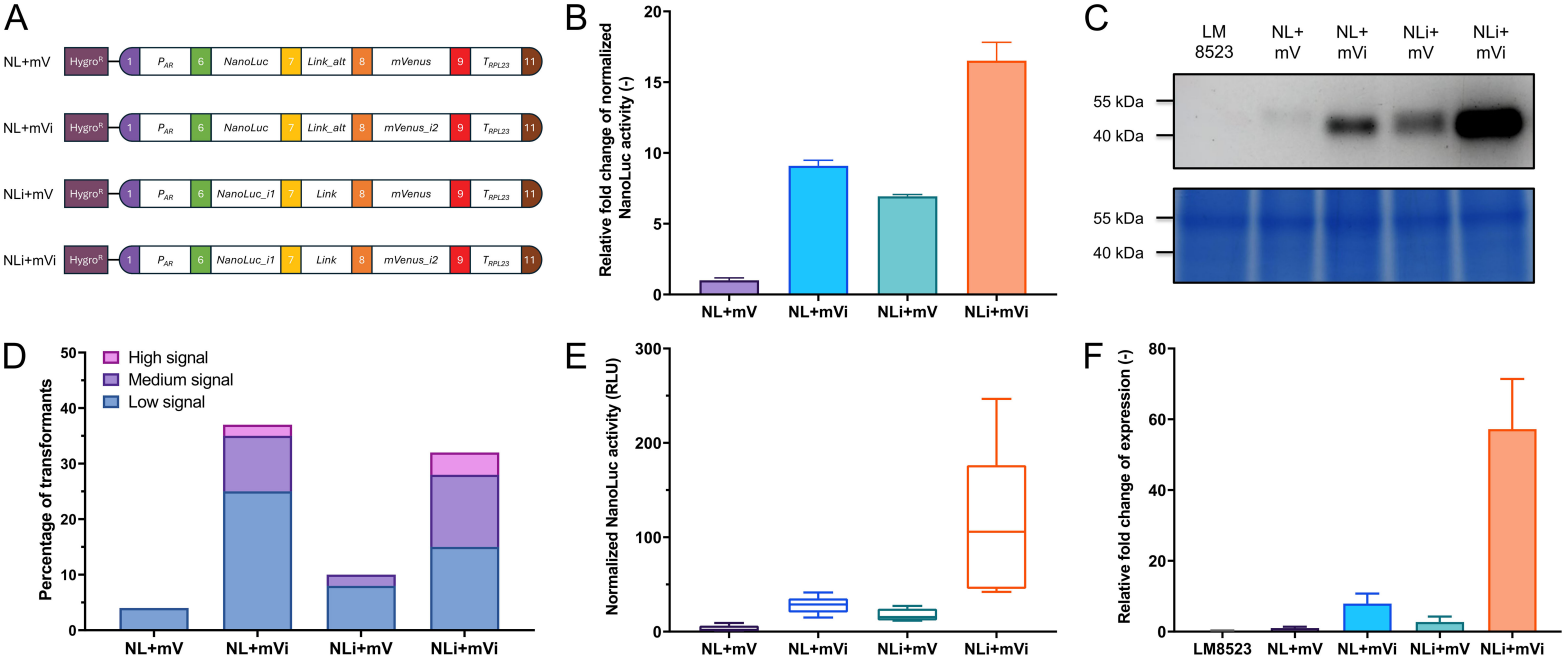
Expression of differently intronized NanoLuc constructs in *C. reinhardtii*. **(A)** Level 2 devices containing a transcriptional unit for NanoLuc-mVenus fusion protein expression and the *aphVII* cassette (HygroR), conferring resistance to hygromycin. P_AR_: *HSP70A-RBCS2* hybrid promoter. Link: GGSGGR linker peptide. Link_alt: Alternative GGSGGR linker peptide, in-frame correction for wrongly designed B3 parts. T_RPL23_: *RPL23* terminator. **(B)** Measurements of NanoLuc activity in relative light units (RLU) from pooled transformants of the four constructs. Relative activity is compared as fold change to construct NL+mV. **(C)** In-gel detection of protein abundance of the NanoLuc fusion proteins (upper subpanel) and Coomassie blue protein loading control of the same polyacrylamide gel (lower subpanel). **(D)** Relative expression levels of the transformant population of each construct, calculated from the NanoLuc activity signal of 160 individual, independent transformants per construct. Signal intensity was categorized on an arbitrary scale. Low signal: Above background signal - 20 A.U.; medium signal: 20 - 99 A.U.; high signal: ≥ 100 A.U. **(E)** Measurements of NanoLuc activity from the five highest expressing lines per construct. **(F)** Quantification of relative expression levels of pools of the five highest expressing transformant lines per construct, as measured by real-time qPCR. Measurements occurred in technical quintuplicates and the fold change to construct NL+mV was calculated. For all NanoLuc activity measurements, individual samples were measured in a technical quadruplicate. The background signal of LM8523 was subtracted from the raw data and measurements were normalized to cell numbers via chlorophyll fluorescence. Error bars in bar charts represent standard deviations from mean.

To demonstrate the functionality of our approach, we have cloned the assembled level 0 parts into expression modules, based on the level 2 backbone vectors described by Niemeyer and Schroda (2022). Firstly, we tested differently intronized *NanoLuc-mVenus* gene fusions that varied in intron insertion site, type, and number and compared them to an intron-less construct (NL+mV) serving as a control (**Figure 3A**). We used NanoLuc due to its properties as a small, highly sensitive luminescent reporter. Their expression was driven by the *HSP70A-RBCS2* (*AR*) promoter and the *RPL23* terminator, a strong promoter-terminator combination reported to result in high expression levels (Einhaus et al., 2021; Kiefer et al., 2022; Niemeyer et al., 2023). After transformation into *C. reinhardtii* LM8523 strain, transgene expression was determined at RNA, protein abundance, and reporter activity level. NanoLuc activity was measured pooling more than 1000 individual transformants per construct and revealed a clear positive correlation between expression strength and intron number (**Figure 3B**). Incorporating RBCS2i2 into the downstream region of the gene fusion increased NanoLuc activity by approximately 6-fold, while adding RBCS2i1 to the upstream region resulted in a 9-fold enhancement compared to the control construct. The simultaneous addition of both introns resulted in a more than 16-fold enhancement of NanoLuc activity over the intron-less module. These results were confirmed at the protein level, where the NanoLuc moiety allowed for direct detection in the acrylamide gel (Li et al., 2021). Fusion proteins of the calculated weight of approximately 50 kDa were detected for all four constructs (**Figure 3C**). Consistent with NanoLuc activity data, the highest signal was observed for NLi+mVi. Both constructs containing one intron displayed moderate expression, while the control exhibited low expression. Conversely, and in contrast to measurements at protein activity and abundance level, no clear difference between the expression strength of pools was observed with respect to mRNA levels (**Supplementary Figure 2A**). We attribute this to the high sensitivity and specificity of the NanoLuc assay in combination with the high percentage of transformants in the pools with no measurable expression, a phenomenon commonly observed when using *C. reinhardtii* for transgene expression (Sproles et al., 2022). Hence, we turned towards analyzing the expression levels of a subset of transformants with measurable NanoLuc expression. Firstly, 160 single transformants were randomly isolated per construct, individually cultured in microwell plates and tested for reporter expression. Across all tested modules, the vast majority of transformants showed no detectable reporter signal (**Figure 3D**). When grouping transformants into an arbitrary scale of signal strength however, it was possible to observe extensive differences between the constructs. For the intron-less control, less than 5% of the tested transformants had a detectable, but weak signal and the fusion gene intronized with RBCS2i1 yielded only around 10% transformants with medium to low activity. NL+mVi and NLi+mVi both generated an overall higher percentage of around one third of successfully expressing colonies, the latter construct yielding a higher number of high and medium expressing clones. Next, we pooled the five top-performing mutant lines per module and measured their NanoLuc activity (**Figure 3E**) and mRNA level (**Figure 3F**), which further confirmed the previous results with a clear correlation this time also at transcript level. Transcript abundance in the pools of the strongest clones was enhanced around 60-fold when inserting the two introns, whereas the insertion of one intron only improved mRNA levels between 2- and 8-fold, depending on intron and insertion site. Taken together, our different experimental approaches show that RBCS2i2 yielded a slightly stronger IME than RBCS2i1 when inserted into the reporter fusion, while the strongest effect was observed when both introns were used simultaneously. Interestingly, no mVenus fluorescence was observed from the fusion protein (data not shown). We attribute this to protein misfolding and thus loss of function.

### 3.4 Expression of a fungal luciferase implements part of a bioluminescence pathway in *C. reinhardtii*

**Figure 4:**
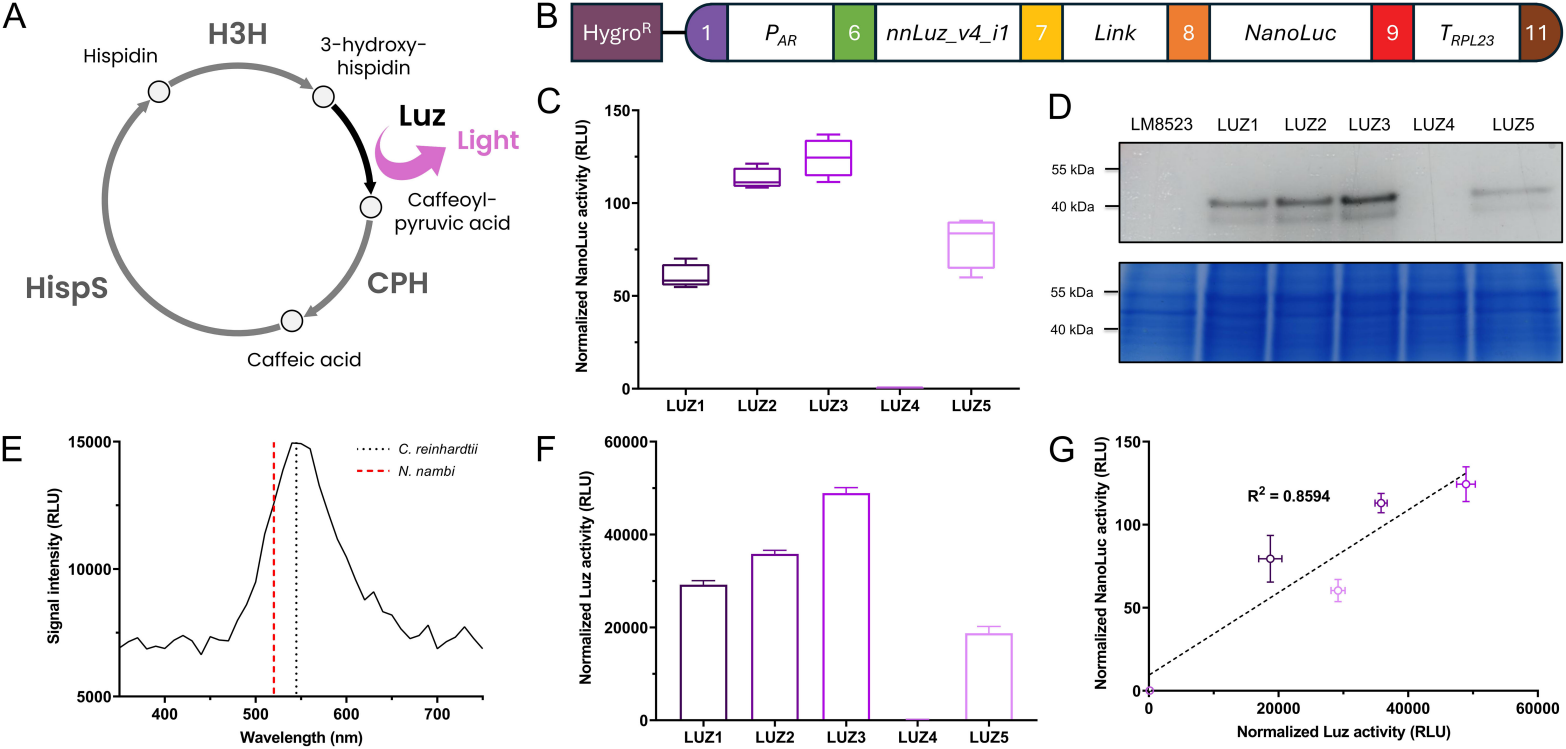
Expression of *Luz* to integrate part of a bioluminescence pathway in *C. reinhardtii*. **(A)** Simplified caffeic acid cycle from *N. nambi* (Kotlobay et al., 2018). 3-hydroxyhispidin is oxidized to caffeoylpyruvic acid under light emission. The intermediate is first converted to caffeic acid and then to hispidin, before being recovered as the original substrate of the cycle. CPH, Caffeoylpyruvate hydrolase; HispS, Hispidin synthase; H3H, Hispidin-3-hydroxylase; Luz, Luciferase. **(B)** Level 2 device containing a transcriptional unit for *Luz* expression and the *aphVII* cassette (HygroR), conferring resistance to hygromycin. **(C)** Measurements of NanoLuc activity in RLU of the five strongest overexpressing transformant lines. **(D)** In-gel detection of protein abundance of the NanoLuc fusion proteins (upper subpanel) and Coomassie blue protein loading control of the same polyacrylamide gel (lower subpanel). **(E)** Emission spectrum of the light produced from Luz by conversion of 3-hydroxyhispidin *in vitro*. The spectrum was recorded in 10 nm steps. Emission maxima of *N. nambi* mycelium [data from Kotlobay et al. (2018)] and the *C. reinhardtii* protein extract are indicated by the dashed and the dotted line, respectively. **(F)** *In vitro* measurements of Luz activity in RLU using protein extracts of the five strongest overexpressing transformants lines. Raw measurements were normalized to the protein concentration of the extracts. **(G)** Correlation between normalized Luz activity and normalized NanoLuc activity, with coefficient of determination (R^2^) for simple linear regression. Measurements of NanoLuc activity were carried out and normalized as previously described. Error bars in the bar chart and the correlation plot represent standard deviations from mean.

To further demonstrate the proper functioning of the assembled level 0 parts, we constructed an expression vector for nnLuz_v4, a novel luciferase with many possible biotechnological applications that had recently been sequence optimized for stability and activity (Shakhova et al., 2024). As an integral part of the caffeic acid cycle that is at the base of fungal bioluminescence in nature, Luz oxidizes the substrate 3-hydroxyhispidin, resulting in the emission of light, before the substrate is recovered via caffeic acid (**Figure 4A**). The expression was controlled via the combination of *AR* promoter and *RPL23* terminator and the gene was fused to *NanoLuc* as a reporter (**Figure 4B**). After screening 96 randomly picked individual colonies, the five strongest expressing clones were selected for further examination. When re-tested for NanoLuc reporter signal, four transformants showed considerable expression, while one lost its transgene expression capabilities, indicating silencing of the transgene in this line (**Figure 4C**). Protein abundance determined via in-gel detection revealed a similar trend and the largest band, most likely representing the 50 kDa fusion protein, supported the observed expression patterns on enzyme activity level (**Figure 4D**). Subsequently, we aimed to measure activity of the Luz moiety of the fusion protein. Proteins were extracted with a commercially available lysis buffer for *Renilla* luciferase. Following extraction, the cell lysate was incubated with the substrate 3-hydroxyhispidin to assess Luz activity. In the assay, the four well-expressing transformant lines showed clear light signals that were largely stable over a period of up to 1 h after substrate addition (**Supplementary Figure 3A**). The silenced transformant LUZ4, as well as the background strain LM8523 did not produce any detectable light signal. Similarly, no signal was observed when protein extracts were incubated with a DMSO mock (**Supplementary Figure 3B**). These results confirm that light emission originates specifically from the conversion of 3-hydroxyhispidin by Luz. Analysis of the emitted light revealed a peak at approximately 540 nm (**Figure 4E**), similarly to that of the light that *N. nambi* mycelium emits (Kotlobay et al., 2018). The measured Luz activity closely aligns with the NanoLuc activity data, where LUZ3 demonstrated the highest normalized activity, followed by LUZ2, LUZ1 and LUZ5 (**Figure 4F**). The strong positive correlation between the light signals of both expressed luciferases of the fusion protein underscores the reliability of NanoLuc as a reporter in this system (**Figure 4G**). Furthermore, it’s an indicator for correct folding of each moiety and verifies that the assay conditions for determining Luz activity were technically correct and no substrate scarcity occurred.

### 3.5 Overexpression of a tryptophan decarboxylase does not result in tryptamine accumulation

As a final demonstration, we constructed plasmids for expression of the tryptophan decarboxylase PsiD. In its native fungal host *P. cubensis*, the enzyme is part of the psilocybin pathway and catalyzes the conversion of tryptophan to tryptamine (Fricke et al., 2017). Due to its indole core, tryptamine is a promising precursor compound for many pharmaceutical drugs and agrochemicals (Milne et al., 2023). We created both an N-terminal and a C-terminal fusion of the enzyme with the reporter NanoLuc (**Supplementary Figure 4A**) and transformed this into *C. reinhardtii* LM8523. After screening 96 randomly selected individual colonies per construct, no transformant line displayed expression levels above the detection threshold. We suspected reporter misfolding in the fusion protein and hence constructed an additional expression plasmid (**Supplementary Figure 4A**). Here, the two gene sequences are separated by the viral 2A peptide CDS, allowing for bicistronic gene expression (Plucinak et al., 2015). Screening of single transformants yielded a number of well-expressing lines (**Supplementary Figure 4B**), supporting the assumption of reporter misfolding in the fusion constructs. We cultivated the five strongest overexpressing lines in TAP and prepared methanol extracts from the resulting culture supernatant and cell pellets, a technique commonly used to extract tryptamine from plant material (Ly et al., 2008). In an HPLC-based quantification attempt, however, we could not observe any accumulation of intra- or extracellular tryptamine (data not shown). As in plants, tryptamine has been identified as an intermediate metabolite in the auxin pathway (Quittenden et al., 2009), we suspected that produced tryptamine might undergo further metabolism, but could not observe accumulation of IAA either (data not shown). To understand possible phenotypic consequences of PsiD expression, the two most potent transformant lines were cultivated in TAP medium until stationary phase was reached and their performance was compared to that of the background strain LM8523 (**Supplementary Figure 4C**). With regards to growth kinetics and final cell densities reached, no apparent difference between the three strains was detected.

## 4 Discussion

Here, we developed MoInClo as a novel intron insertion system based on the MoClo syntax for optimized transgene expression in the green alga *C. reinhardtii*. Our system is fully compatible with existing MoClo standardized parts, thus allowing integration into automated platforms like RoboMoClo (Kang et al., 2022). Such automation, when implemented in biofoundries, has vast potential to accelerate the design-build-test-learn cycle of synthetic biology and boost biomolecule production in *C. reinhardtii*. Moreover, the system is not restricted to the alga, but can be adapted to virtually any expression system with an existing MoClo kit with no further challenges anticipated for adapting it. As IME is a phenomenon that is observed in various organisms used for protein expression, including plants, yeasts, insects, and mammals (Laxa, 2017), the implications of this work extend well beyond *Chlamydomonas*.

Research in higher plants indicates that IME is caused by the key role introns play in DNA transcription, mRNA stability, and its export from the nucleus, resulting in generally increased mRNA levels when introns are incorporated into genes (Laxa, 2017; Rose, 2019; Shaul, 2017). Positive effects on mRNA translation and chromatin accessibility have also been identified in other organisms (Gallegos and Rose, 2015; Hoshida et al., 2017; Karve et al., 2011). In yeast, intron-dependent gene looping was observed, where the intron physically interacts with both promoter and terminator (Moabbi et al., 2012), indicating that similar mechanisms might be at play during IME in *C. reinhardtii*. The interactions between introns and other elements of genetic regulation might explain the somewhat contradictory results found when using the same intron but different promoter-terminator pairs for transgene expression in *C. reinhardtii* (Baier et al., 2020; Ferrante et al., 2008; Lumbreras et al., 1998). This highlights the need to systematically test interactions between introns and other genetic control elements to achieve a predictable output for synthetic biology applications. MoInClo can facilitate this process by enabling flexible intron insertion into the same transgene, thus allowing to clone a wide variety of different expression constructs with several combinations of promoters, introns, and terminators. For this purpose, our system offers high flexibility without compromising the complexity of the expression constructs when compared to the intron parts that are supplied with the *Chlamydomonas* MoClo toolkit (Crozet et al., 2018), as well as the traditional way of intron insertion into coding sequences via synthesizing the fully intronized gene entirely.

We substantially demonstrate the feasibility of the cloning approach by assembling several genes with different introns in various insertion sites. It performs well, successfully assembling up to seven disparate fragments without requiring extensive colony screening to identify the correctly constructed plasmid. During assemblies of level 0 parts, we used level -1 introns throughout this study. In a less laborious manner, it is also possible to use the introns directly as synthetic DNA fragments. However, this approach comes at the cost of a somewhat reduced assembly efficiency. Due to errors occurring in DNA synthesis, the quality of gene fragments is significantly lower compared to sequence-verified, purified plasmids. As level -1 intron cloning has a high success rate, can be easily parallelized for many introns and the final intron plasmid can be reused for other assemblies, we recommend taking this route when using our system. The increased flexibility of our system comes with the tradeoff of higher fragment numbers in the assembly. In our case, this resulted in a seven-fragment assembly for *PsiD*, compared to what would have been a three-fragment assembly with a conventional approach (Schroda, 2019). This increases the chance of potential assembly errors and complicates cloning, a problem that we aim to circumvent by supplying the backbone vectors pICH41258_3A and pICH41258_3B for large genes to be split in 2 parts. A further challenge associated with our system lies in the design of overhangs and restriction enzyme recognition sites prior to gene synthesis. Successful implementation of this approach requires a thorough understanding of IME in *C. reinhardtii*, as well as proficiency in Golden Gate cloning techniques. These requirements can make the gene design process both complex and time-consuming. This limitation could be addressed by the development of a web service to streamline the design process of gene fragments for synthesis. The creation of this application represents a promising direction for future development, potentially enhancing the utility and adoption of our method.

After successful level 0 cloning, we demonstrate the functionality of the resulting parts by cloning expression modules and transforming them in LM8523. In the following, we aim to discuss the findings of these experiments, as they give insights into transgene design for successful expression in *C. reinhardtii* and show the potential of algae-based bioprocesses.

Throughout this study, we used *C. reinhardtii* LM8523 due to its nonfunctional *SRTA* allele that grants superior transgene expression (Neupert et al., 2020). Thus far, however, only one previous work used the strain for recombinant protein production (Kiefer et al., 2022). In our hands, transforming a well-intronized expression module into LM8523 yielded a third of transformant colonies with measurable levels of reporter expression and sufficient total numbers of highly expressing lines. This percentage is comparable to studies using UVM4 for transgene expression of monocistronic constructs (Baier et al., 2018, 2020; Jacobebbinghaus et al., 2024). Our results highlight the robust transgene expression capabilities of LM8523, positioning it as a strong alternative to the widely used UVM4. The functional cell wall of LM8523 makes it an attractive choice for biotechnological applications where more sturdy cellular features are desired. It has to be noted that all characterization experiments in this work were carried out within a maximum time of two months after transformation of the respective construct and therefore no statement can be made regarding the long-term stability of transgene expression in this strain.

During the experiments with different *NanoLuc-mVenus* constructs, we were unable to detect any fluorescent signal, despite observing luciferase activity with these constructs. This may be due to a possible misfolding in the C-terminal part of the fusion protein, a phenomenon often observed when expressing fluorescent proteins as gene fusions (Pédelacq et al., 2006). This might hinder correct formation of the active site, even though a previously validated flexible linker was used to separate the moieties of the fusion proteins (Kasai and Harayama, 2016). Similar phenomena were seen when expressing the *NanoLuc-PsiD* and *PsiD-NanoLuc* gene fusions, where neither the C-terminal reporter connected by a flexible linker, nor the N-terminal, directly fused reporter generated any detectable NanoLuc signal in the tested colonies. The usage of the F2A peptide, resulting in two separate proteins instead of a fusion protein (Plucinak et al., 2015), solved the problem, supporting our assumption of protein misfolding as the primary cause of the observed phenomena.

When comparing expression of differently intronized constructs, we observed a clear positive correlation between intron numbers within a transgene and its expression level, consistent with findings from previous studies (Baier et al., 2018, 2020). Interestingly, the addition of RBCS2i2 to the downstream part of the *NanoLuc-mVenus* gene fusion resulted in stronger IME than the addition of RBCS2i1 to its upstream part. These results stand in contrast to the findings of Jaeger et al. (2019) and Baier et al. (2020), who reported much higher levels of IME for RBCS2i1 when either of the two introns was inserted into the same position of a reporter plasmid. Our findings are further contrasted when taking into consideration the intron insertion site of RBCS2i1 (AG/GT) that according to the systematic studies of Baier et al. (2018) should yield a stronger IME than the insertion site for RBCS2i2 (CG/GT). This indicates that the position of the intron within a coding sequence can strongly influence its level of IME, possibly more than its sequence determinants or neighboring nucleotides. Further systematic research is needed in order to unveil the impact of the intron insertion site within the CDS on transgene expression in *C. reinhardtii*.

The successful expression of nnLuz in *C. reinhardtii*, enabling the first step of the caffeic acid cycle that underlies fungal bioluminescence, is a promising start for the implementation of the full cycle in a microbial photosynthetic chassis. Fully realized and optimized, we envision the possibility to use it as an environmentally sustainable lighting solution, potentially replacing artificial light sources in urban environments. Such systems powered by photosynthesis could minimize the energy footprint associated with lighting technology in the future. Furthermore, the high signal-to-noise ratio observed in well-expressing transformants highlights the potential of nnLuz for future applications as a reporter, comparable to established systems such as *Renilla* luciferase and NanoLuc. In our experiments, the detected bioluminescence emission maximum of 540 nm differed slightly from that of the native host of the caffeic acid cycle, *N. nambi*, that had an emission peak around 520 nm (Kotlobay et al., 2018). This is conceivably caused by differences in pH and salt concentrations between the buffer used in our experiment and the cellular environment of *N. nambi*. Such factors have been shown to influence the emission spectrum of other luciferases and are likely the cause for the phenomenon observed in our study (Viviani et al., 2022). These properties of luciferases can be exploited in the future by enabling the color-tuning of Luz for specific applications.

In our hands, the overexpression of the tryptophan decarboxylase PsiD did not result in intracellular tryptamine accumulation or its enrichment in the growth medium. In plants, tryptamine contributes to IAA biosynthesis (Quittenden et al., 2009), and a recent study suggests that similar genes to those involved in IAA biosynthesis in plants also play a role in *C. reinhardtii* (De Marco et al., 2024). Nevertheless, we did not observe IAA accumulation either. As tryptamine serves as an intermediate compound in the metabolism of many plants (Negri et al., 2021), it is conceivably further metabolized also in *C. reinhardtii* and thus does not accumulate inside the cell. Due to the F2A peptide used in the expression construct, PsiD dysfunction through protein misfolding is unlikely. Other factors though, such as incompatibility of PsiD with the cellular environment of *C. reinhardtii* might explain the absence of accumulated tryptamine. If successfully achieved, the overexpression of tryptophan decarboxylases in *C. reinhardtii* could enable the alga to serve as an environmentally sustainable chassis for the production of pharmaceutically relevant tryptamines like psilocybin or N,N-DMT, compounds with vast therapeutic potential (McClure-Begley and Roth, 2022; Nutt et al., 2023).

## Data Availability

The original contributions presented in the study are included in the article/**Supplementary Material**. Further inquiries can be directed to the corresponding author.
