# Supplementary material for: A novel MoClo-mediated intron insertion system facilitates enhanced transgene expression in Chlamydomonas reinhardtii
Source: Front Plant Sci. 2025 Mar 7;16:1544873. doi: 10.3389/fpls.2025.1544873 (PMC11925875; doi:10.3389/fpls.2025.1544873)
Supplement: Supplementary file 1 [file DataSheet1.docx]

Supplementary Material

# Supplementary Figures

Supplementary Figure 1: Schematic description of the influence of the intron insertion site in a gene on splicing efficiency in *C. reinhardtii* (Data from Baier et al., 2018). (A) Influence of the first nucleotide at the 5’ and the 3’ splice site outside the intron. (B) Influence of the first two nucleotides at the 5’ and the 3’ splice site outside the intron, with the first nucleotide being guanine for both sites.

Supplementary Figure 2: Determination of expression levels of the four *NanoLuc-mVenus* constructs by real-time qPCR and primer validation. (A) Quantification of relative expression levels of pooled transformants of the constructs, as measured by real-time qPCR. Measurements occurred in technical triplicates and the fold change to construct NL+mV was calculated. (B) Amplification efficiency of qPCR primers qPCR_mVenus_F and qPCR_mVenus_R. The slope of -3.3079 indicates an amplification efficiency of 100.59 %.

Supplementary Figure 3: *In vitro* measurements of Luz activity over time, using protein extracts of the five strongest overexpressing transformants lines and the wild-type LM8523 incubated with (A) the substrate 3-hydroxyhispidin or (B) DMSO as a mock. The first 15 measurements were taken every minute, the remainder of measurement every 5 min.

Supplementary Figure 4: Expression of the tryptophan decarboxylase PsiD in *C. reinhardtii*. (A) Level 2 devices containing a transcriptional unit for *PsiD* expression and the *aphVII* cassette (HygroR), conferring resistance to hygromycin. (B) Measurements of NanoLuc activity in RLU of the five strongest overexpressing transformant lines, carried out and normalized as previously described. (C) Growth curve of the background strain LM8523, as well as PSID1 and PSID2 as the two strongest overexpressing lines in TAP medium.

# Supplementary tables

Supplementary Table 1: Oligos used for cloning.

| **Name** | **Sequence (5’ -> 3’)** | **Cloning** |
| --- | --- | --- |
| Gibson_DraI-BsaI_F | taatgtactggggtggatgcagtgggccccactctgccgaattcggatccggagtg | pCM-1 |
| Gibson_DraI-BsaI_R | Atatcctgtcaaacactgatagtttaaaccacttcgcagcgtgagaccgtcacag | pCM-1 |
| pMC_3A_ACGA_3F | cgaagacaaACGAtgagaccacgaagtggctcttcag | pICH41258_3A and B, Fragment 1 |
| Hsp_Gibson_R | ACGGGGTCTGACGCTCAGTG | pICH41258_3A, Fragment 1 |
| pMC_A3_ACGA_3R | tctcaTCGTttgtcttcgtcacagcttgtctgtaagc | pICH41258_3A and B, Fragment 2 |
| Hsp_Gibson_F2 | cactgagcgtcagaccccgt | pICH41258_3A, Fragment 2 |
| pMC_3B_ACGA_5F | gtggtctcaACGAttgtcttcgcagctggcacgacagg | pICH41258_3B |
| pMC_B3_ACGA_5R | gacaaTCGTtgagaccacagagtgattaatgaatcggccaacgc | pICH41258_3B |
| Ann_Stop_F | ACGGAAGACttAGGTTAAGCTTaaGTCTTCACG | pCM0-Stop (B4-B5) |
| Ann_Stop_R | CGTGAAGACttAAGCTTAACCTaaGTCTTCCGT | pCM0-Stop (B4-B5) |
| Anneal_GGSGGR_alt_linker_B4_F | ACGGAAGACttAGGTCCGGCGGCAGCGGCGGGCGCCCTTCGaaGTCTTCAGC | pCM0-GGSGGR_alt (B4) |
| Anneal_GGSGGR_alt_linker_B4_R | GCTGAAGACttCGAAGGGCGCCCGCCGCTGCCGCCGGACCTaaGTCTTCCGT | pCM0-GGSGGR_alt (B4) |
| GGSGGR_B4_F | GAAGACttAGGTGGCGGCAGCGGCGGGCGCCCTTCGaaGTCTTC | pCM0-GGSGGR (B4) |
| GGSGGR_B4_R | GAAGACttCGAAGGGCGCCCGCCGCTGCCGCCACCTaaGTCTTC | pCM0-GGSGGR (B4) |

Supplementary Table 2: Gene fragment sequences.

| **Name** | **Sequence (5’ -> 3’)** | **Cloning** |
| --- | --- | --- |
| Fragment_PsiD_1 (B3) | tggcaggatatattgtggtgtaaacGAAGACttAATGcaggtgatccccgcctgcaacagcgccgccatccgcagcctgtgccccacccccgagagcttccgcaacatgggctggctgagcgtgagcgacgccgtgtacagcgagttcatcggcgagctggccacccgcgccagcaaccgcaactacagcaacgagttcggcctgatgcagcccatccaggagttcaaggccttcatcgagagcgaccccgtggtgcaccaggagttcatcgacatgttcgagggcatccaggacagcccccgcaactaccaggagctgtgcaacatgttcaacgacatcttccgcaaggcccccgtgtacggcgacctgggcccccccgtgtacatgatcatggccaagctgatgaacacccgcgccgaaGTCTTCtgacaggatatattggcgggtaaac | pCM0-PsiD_i1 (B3) |
| Fragment_PsiD_2 (B3) | tggcaggatatattgtggtgtaaacGAAGACttgcttcagcgccttcacccgccagcgcctgaacctgcacttcaagaagctgttcgacacctggggcctgttcctgagcagcaaggacagccgcaacgtgctggtggccgaccagttcgacgaccgccactgcggctggctgaacgagcgcgccctgagcgccatggtgaagcactacaacggccgcgccttcgacgaggtgttcctgtgcgacaagaacgccccctactacggcttcaacagctacgacgacttcttcaaccgccgcttccgcaaccgcgacatcgaccgccccgtggtgggcgaaGTCTTCtgacaggatatattggcgggtaaac | pCM0-PsiD_i1 (B3) |
| Fragment_PsiD_3 (B3) | tggcaggatatattgtggtgtaaacGAAGACttgcgtgaacaacaccaccctgatcagcgccgcctgcgagagcctgagctacaacgtgagctacgacgtgcagagcctggacaccctggtgttcaagggcgagacgtacagcctgaagcacctgctgaacaacgaccccttcaccccccagttcgagcacggcagcatcctgcagggcttcctgaacgtgaccgcctaccaccgctggcacgcccccgtgaacggcaccatcgtgaagatcatcaacgtgcccggcacctacttcgcccaggcccccagcaccatcggcgaccccatccccgacaacgactacgacccccccccctacctgaagagcctggtgtacttcagcaacatcgccgcccgccagatcatgttcatcgaggccgacaacaagaaGTCTTCtgacaggatatattggcgggtaaac | pCM0-PsiD_i1 (B3) |
| Fragment_PsiD_4 (B3) | tggcaggatatattgtggtgtaaacGAAGACttgagatcggcctgatcttcctggtgttcatcggcatgaccgagatcagcacctgcgaggccaccgtgagcgagggccagcacgtgaaccgcggcgacgacctgggcatgttccacttcggcggcagcagcttcgccctgggcctgcgcaaggactgccgcgccgagatcgtggagaagttcaccgagcccggcaccgtgatccgcatcaacgaggtggtggccgccctgaaggccggAGGTaaGTCTTCtgacaggatatattggcgggtaaac | pCM0-PsiD_i1 (B3) |
| Fragment_Luz_1 (B3) | tggcaggatatattgtggtgtaaacGAAGACttAATGcgcagcaccatcagcctgagcagcctgctggagcgcctgagcaagctgagcagccgcagcatcgccatcacctgcggcgtggtgctggccagcgccatcgccttccccatcatccgccgcgactaccagaccttcctggaggtgggccccagctacgccccccagaacttccgcggctacatcaccgtgtgcgtgctgagcctgttccgccaggagcagaagggcctggccatctacgaccgcctgcccgagaagcgccgctggctggccgacctgcccttccgcgagggcccccgccccagcatcaccagccacatcatccagcgccagcgcacccagctggtggaccaggagttcgccacccgcgagctgatcgacaagaaGTCTTCtgacaggatatattggcgggtaaac | pCM0_Luz_v4_i1 (B3) |
| Fragment_Luz_2 (B3) | tggcaggatatattgtggtgtaaacGAAGACttgtgatcccccgcgtgcaggcccgccacaccgacaagaccttcctgagcaccagcaagttcgagttccacgccaaggccatcttcctgctgcccagcatccccatcaacgaccccctgaacatccccagccacgacaccgtgcgccgcaccaagcgcgagatcgcccacatgcacgactaccacgactgcagcctgcacctggccctggccccccaggacggcaaggaggtgctgaagaagggctggggccagcgccaccccctggccggccccggcgtgcccggcccccccaccgagtggaccttcctgtacgccccccgcaacgaggaggaggcccgcgtggtggagatgatcgtggaggccagcatcggctacatgaccaacgaccccgccggcaagatcgtggagaacgccggAGGTaaGTCTTCtgacaggatatattggcgggtaaac | pCM0_Luz_v4_i1 (B3) |
| Fragment_NanoLuc_1 (B3) | tggcaggatatattgtggtgtaaacGAAGACttAATGGTGTTCACCCTGGAGGACTTCGTGGGCGACTGGCGCCAGACCGCCGGCTACAACCTGGACCAGGTGCTGGAGCAGGGCGGCGTGAGCAGCCTGTTCCAGAACCTGGGCGTGAGCGTGACCCCCATCCAGCGCATCGTGCTGAGCGGCGAGAACGGCCTGAAGATCGACATCCACGTGATCATCCCCTACGAGGGCCTGAGCGGCGACCAGATGGGCCAGATCGAGAAGATCTTCAAGGTGGTGTACCCCGTGGACGACCACCACTTCAAGaaGTCTTCtgacaggatatattggcgggtaaac | pCM0_NanoLuc_i1 (B3) |
| Fragment_NanoLuc_2 (B3) | tggcaggatatattgtggtgtaaacGAAGACttGTGATCCTGCACTACGGCACCCTGGTGATCGACGGCGTGACCCCCAACATGATCGACTACTTCGGCCGCCCCTACGAGGGCATCGCCGTGTTCGACGGCAAGAAGATCACCGTGACCGGCACCCTGTGGAACGGCAACAAGATCATCGACGAGCGCCTGATCAACCCCGACGGCAGCCTGCTGTTCCGCGTGACCATCAACGGCGTGACCGGCTGGCGCCTGTGCGAGCGCATCCTGGCCGGAGGTaaGTCTTCtgacaggatatattggcgggtaaac | pCM0_NanoLuc_i1 (B3) |
| Fragment_NanoLuc_1 (B5) | tggcaggatatattgtggtgtaaacGAAGACttTTCGGTGTTCACCCTGGAGGACTTCGTGGGCGACTGGCGCCAGACCGCCGGCTACAACCTGGACCAGGTGCTGGAGCAGGGCGGCGTGAGCAGCCTGTTCCAGAACCTGGGCGTGAGCGTGACCCCCATCCAGCGCATCGTGCTGAGCGGCGAGAACGGCCTGAAGATCGACATCCACGTGATCATCCCCTACGAGGGCCTGAGCGGCGACCAGATGGGCCAGATCGAGAAGATCTTCAAGGTGGTGTACCCCGTGGACGACCACCACTTCAAGaaGTCTTCtgacaggatatattggcgggtaaac | pCM0_NanoLuc_i2 (B5) |
| Fragment_NanoLuc_2 (B5) | tggcaggatatattgtggtgtaaacGAAGACttGTGATCCTGCACTACGGCACCCTGGTGATCGACGGCGTGACCCCCAACATGATCGACTACTTCGGCCGCCCCTACGAGGGCATCGCCGTGTTCGACGGCAAGAAGATCACCGTGACCGGCACCCTGTGGAACGGCAACAAGATCATCGACGAGCGCCTGATCAACCCCGACGGCAGCCTGCTGTTCCGCGTGACCATCAACGGCGTGACCGGCTGGCGCCTGTGCGAGCGCATCCTGGCCTAAGCTTaaGTCTTCtgacaggatatattggcgggtaaac | pCM0_NanoLuc_i2 (B5) |
| Fragment_RbcS2_i1_001 | ggtctcaaatgGAAGACttcaagGTGAGTCGACGAGCAAGCCCGGCGGATCAGGCAGCGTGCTTGCAGATTTGACTTGCAACGCCCGCATTGTGTCGACGAAGGCTTTTGGCTCCTCTGTCGCTGTCTCAAGCAGCATCTAACCCTGCGTCGCCGTTTCCATTTGCAGgtgaaaGTCTTCaggttgagacc | pCM-1_i1_001 |
| Fragment_RbcS2_i1_002 | ggtctcaggagGAAGACttcccgGTGAGTCGACGAGCAAGCCCGGCGGATCAGGCAGCGTGCTTGCAGATTTGACTTGCAACGCCCGCATTGTGTCGACGAAGGCTTTTGGCTCCTCTGTCGCTGTCTCAAGCAGCATCTAACCCTGCGTCGCCGTTTCCATTTGCAGgccgaaGTCTTCcgcttgagacc | pCM-1_i1_002 |
| Fragment_RbcS2_i1_003 | ggtctcaggagGAAGACttctcgGTGAGTCGACGAGCAAGCCCGGCGGATCAGGCAGCGTGCTTGCAGATTTGACTTGCAACGCCCGCATTGTGTCGACGAAGGCTTTTGGCTCCTCTGTCGCTGTCTCAAGCAGCATCTAACCCTGCGTCGCCGTTTCCATTTGCAGgcacaaGTCTTCcgcttgagacc | pCM-1_i1_003 |
| Fragment_RbcS2_i1_004 | ggtctcaggagGAAGACtttacgGTGAGTCGACGAGCAAGCCCGGCGGATCAGGCAGCGTGCTTGCAGATTTGACTTGCAACGCCCGCATTGTGTCGACGAAGGCTTTTGGCTCCTCTGTCGCTGTCTCAAGCAGCATCTAACCCTGCGTCGCCGTTTCCATTTGCAGgcgtaaGTCTTCcgcttgagacc | pCM-1_i1_004 |
| Fragment_RbcS2_i2_001 | ggtctcaggagGAAGACttcaaggtgagcttgcggggttgcgagcaacactccagcaacgaacagtgcccaagtcaggaatctgcagtcagcctgggctttcggcggctttttcttgggcaaacagcttgcactcatgccagcgcggcttgtccagcctcacttgagctttccagctgctaccagccgggctatacgacagcgacagagccatagcgtggaatcacttatttgggttgccgaagtagcggtcggagcgtgagttcttggtcaagccgccccttatccggttcctgtccgtgtctttgtccctcgttcacccttcgcggcacccttcatccccttgcttgcaggtgaaaGTCTTCcgcttgagacc | pCM-1_i2_001 |

Supplementary table 3: MoClo level 0 parts used in this study.

| **Name** | **Position** | **Used for** | **Source** |
| --- | --- | --- | --- |
| pCM0-011-pAR | A1-B1 | NL+mV, NL+mVi, NLi+mV, NLi+mVi, Luz_v4, PsiD-NL, PsiD_F2A | Crozet et al., 2018 |
| pCM0-017-pAR | A1-B2 | NL-PsiD | Crozet et al., 2018 |
| pCM0-042-CrNanoLuc | B2 | NL-PsiD | Crozet et al., 2018 |
| pCM0-061-CrNanoLuc | B3 | NL+mV, NL+mVi | Crozet et al., 2018 |
| pCM0-087-2A | B4 | PsiD_F2A | Crozet et al., 2018 |
| pCM0-103-mVenus | B5 | NL+mV, NLi+mV | Crozet et al., 2018 |
| pCM0-104-CrNanoLuc | B5 | Luz_v4 | Crozet et al., 2018 |
| pCM0-113-mVenus(i2) | B5 | NL+mVi, NLi+mVi | Crozet et al., 2018 |
| pCM0-119-tRPL23 | B6-C1 | NL+mV, NL+mVi, NLi+mV, NLi+mVi, Luz_v4, NL-PsiD, PsiD-NL, PsiD_F2A | Crozet et al., 2018 |
| pCM0-NanoLuc_i1 | B3 | NL+mV, NL+mVi | This study |
| pCM0-nnLuz_v4_i1 | B3 | Luz_v4 | This study |
| pCM0-PcPsiD_i1 | B3 | NL-PsiD, PsiD-NL, PsiD-F2A | This study |
| pCM0-Link | B4 | NLi+mV, NLi+mVi, Luz_v4, PsiD-NL | This study |
| pCM0-Link_alt | B4 | NL+mV, NL+mVi | This study |
| pCM0-Stop | B4-B5 | NL-PsiD | This study |
| pCM0-NanoLuc_i2 | B5 | PsiD-NL, PsiD-F2A | This study |
