# Supplementary figures and images for: A novel MoClo-mediated intron insertion system facilitates enhanced transgene expression in Chlamydomonas reinhardtii
Source: Front Plant Sci. 2025 Mar 7;16:1544873. doi: 10.3389/fpls.2025.1544873 (PMC11925875; doi:10.3389/fpls.2025.1544873)

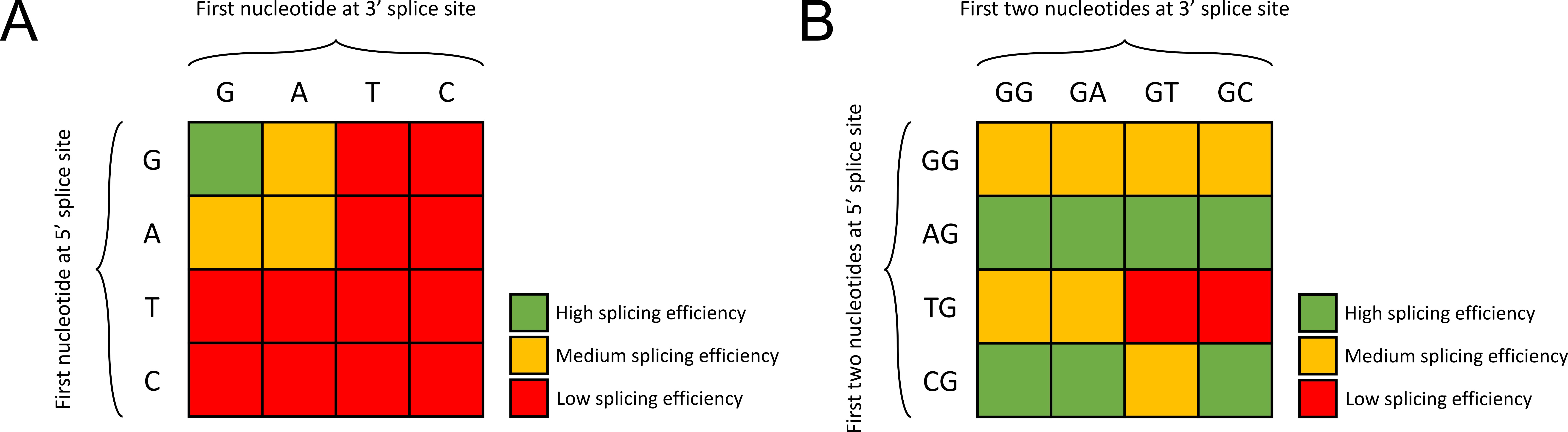

Supplement: Supplementary file 2 [file Image1.jpg]

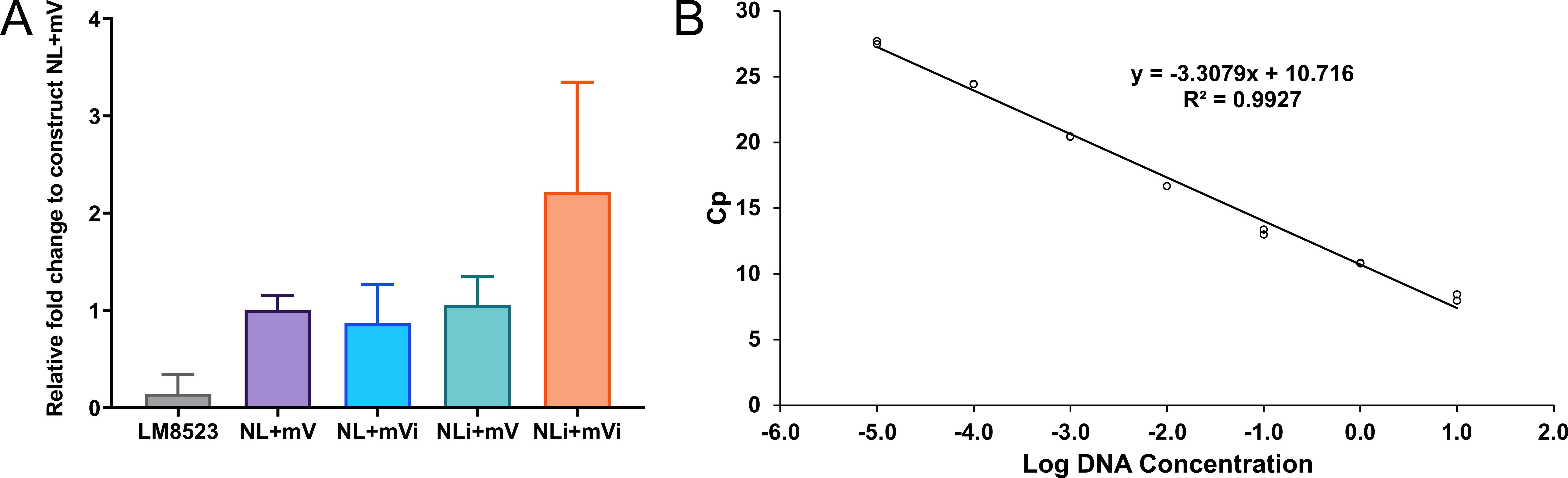

Supplement: Supplementary file 3 [file Image2.jpg]

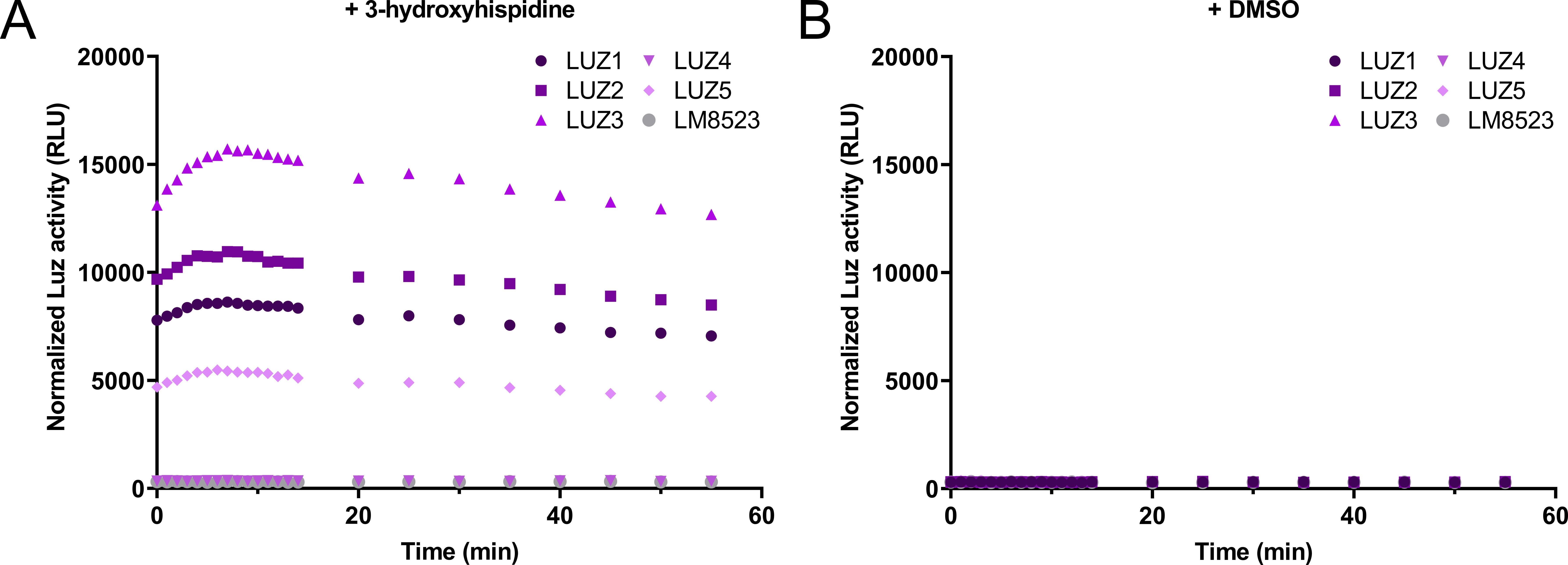

Supplement: Supplementary file 4 [file Image3.jpg]

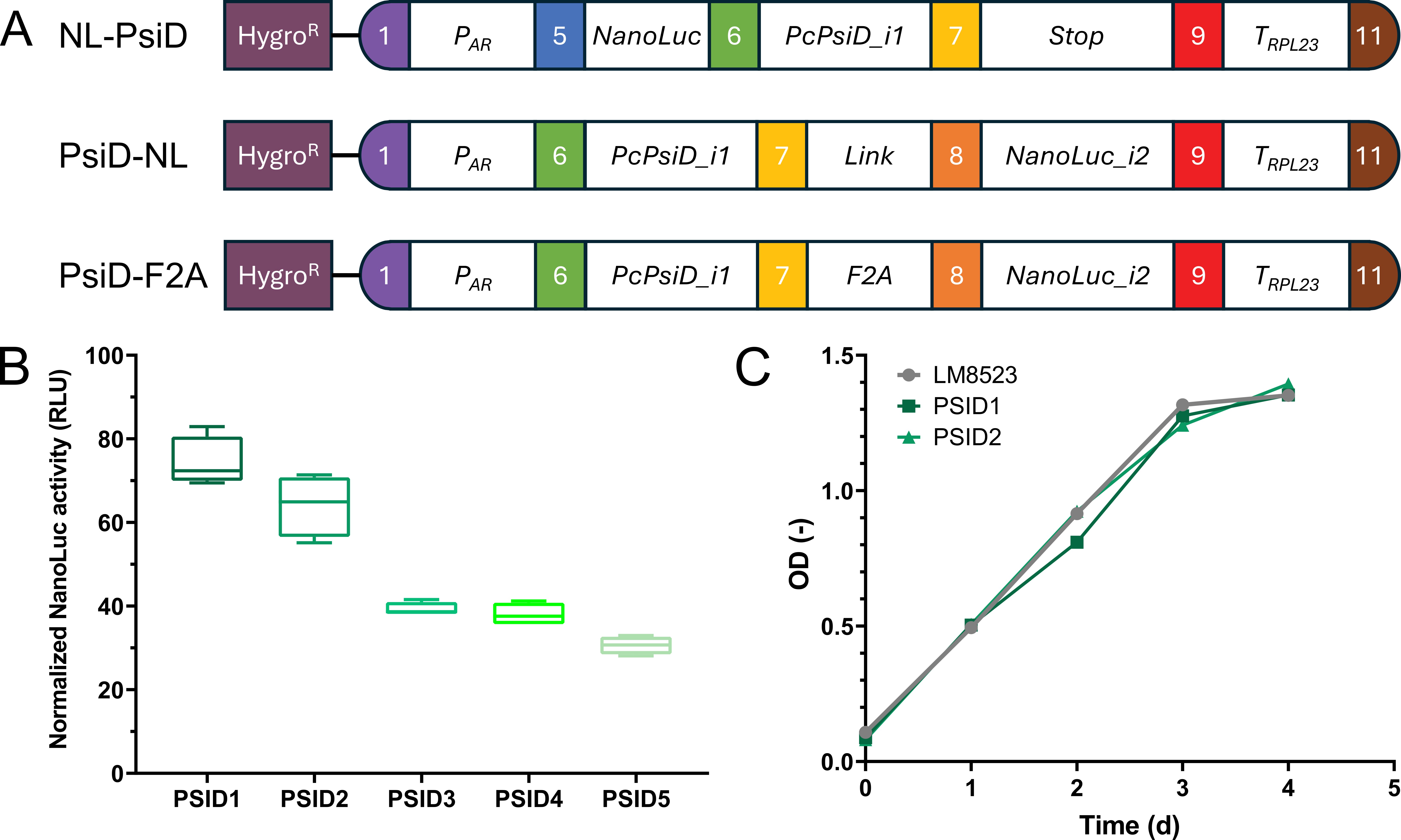

Supplement: Supplementary file 5 [file Image4.jpg]
